# A quasi-experimental study of the video-based “Did You Know?” blended learning model in physical education: Impact on motor skills, knowledge acquisition, and motivation in gymnastics

**DOI:** 10.3389/fpsyg.2026.1695403

**Published:** 2026-05-26

**Authors:** Ahmed Ghorbel, Mohamed Yaakoubi, Mohammed Issa Alsaeed, Noureddine Ben Said, Liwa Masmoudi, Swantje Scharenberg, Adnene Gharbi, Omar Trabelsi

**Affiliations:** 1 Research Unit: Physical Activity, Sport and Health, UR18JS01, National Observatory of Sport, Tunis, Tunisia; 2 The High Institute of Sport and Physical Education, University of Jendouba, Kef, Tunisia; 3 Department of Biomechanics & Motor Behavior, College of Sport Science & Physical Activity, King Saud University, Riyadh, Saudi Arabia; 4 The High Institute of Sport and Physical Education, University of Sfax, Sfax, Tunisia; 5 Institute of Sports and Sports Science, Karlsruhe Institute of Technology, Karlsruhe, Germany; 6 Department of Education Sciences, The High Institute of Applied Studies in Humanities of Mahdia, University of Monastir, Mahdia, Tunisia

**Keywords:** Facebook, gymnastics, hybrid learning, motor learning, multimedia learning, social media

## Abstract

The “Did You Know? with Video” (DYKV) model introduces an innovative blended learning approach that delivers short educational videos via Facebook to optimize learning experiences in physical education (PE), specifically in gymnastics. In a six-week quasi-experimental design, 54 students (Mage = 17 ± 0.40 years) were assigned to either an experimental group (DYKV, *n* = 27) or a control group (traditional approach, TA, *n* = 27). The two groups participated in a 6-week gymnastics unit, which included a 2-h learning session weekly. The DYKV group received hybrid instruction combining online educational media (two videos per week, totaling 12 videos covering different gymnastic elements) shared via Facebook with in-person teaching while the TA group followed a conventional in-person method. Data on motor performance, knowledge retention, and motivation were collected before and after the intervention. The results showed that the DYKV group achieved significantly greater improvements in motor performance (*Δ*%DYKV = +76.69% vs. Δ%TA = +37.58%; *p* < 0.001, η_p_^2^ = 0.124) and knowledge retention (Δ%DYKV = +49.89% vs. Δ%TA = +26.53%; *p* < 0.001, η_p_^2^ = 0.51) alongside a marked increase in autonomous motivation (*p* < 0.001, *r* = 0.73) and decrease in controlled motivation (*p* < 0.001, *r* = 0.87) compared to the TA group. The DYKV model demonstrates potential for enhancing motor, cognitive, and motivational outcomes in PE, further supporting the need for continuous innovation in blended learning within this educational context.

## Introduction

The COVID-19 pandemic precipitated an unprecedented acceleration in the digital transformation of education (Unesco, 2020), compelling institutions worldwide to rapidly adopt blended learning (BL) models that synergize traditional pedagogical approaches with emergent digital technologies (Mhlanga and Moloi, 2020). This global shift has proven particularly consequential for physical education (PE), a discipline historically rooted in face-to-face instruction and practical demonstration (Varea and González-Calvo, 2021). The forced transition to BL revealed both challenges and unexpected opportunities, fundamentally altering our understanding of how motor skills can be effectively taught and acquired in digital-enhanced environments (Potdevin et al., 2018). Research has demonstrated that well-designed BL environments not only maintain educational quality but can significantly enhance student engagement, achievement, and skill retention in PE contexts (Wang et al., 2022; Wang et al., 2023).

Contemporary research has identified four primary BL models that demonstrate particular efficacy in PE settings (Hinojo Lucena et al., 2019). The flipped classroom approach, where students engage with instructional content before in-person practice sessions (Chiang et al., 2019), has shown remarkable success in optimizing limited class time for hands-on skill development (Ghorbel et al., 2024). Rotation models, which alternate between digital and physical learning stations (Kömür et al., 2023), offer opportunities for differentiated instruction and personalized feedback. Flexible models emphasizing self-paced online learning with optional coaching (Priya and Gowrishankar, 2022) have proven effective for learners, while enriched virtual models combining primarily online instruction with targeted in-person sessions (Liu et al., 2022) provide valuable alternatives for schools with limited facilities.

The benefits of BL in PE extend across multiple domains of learning and development. Meta-analytic studies reveal medium-to-large effect sizes for motor skill acquisition in BL environments compared to traditional instruction (Chen et al., 2023). From a motivational perspective, BL environments have been shown to support the psychological needs for autonomy, competence, and relatedness that form the core of self-determination theory (Deci and Ryan, 2013), while simultaneously reducing performance anxiety through asynchronous practice opportunities (Standage and Gillison, 2007).

Despite these demonstrated benefits, significant challenges and inconsistencies persist in the implementation of BL for PE. The transfer of skills from digital learning to applied performance remains inconsistent across studies (Wang et al., 2022; Syafii et al., 2024), suggesting that not all BL approaches equally facilitate this critical transition. Cognitive load considerations present another challenge, as poorly designed digital tools may overwhelm learners’ working memory capacity rather than enhance understanding (Sweller, 2020). Motivational dynamics require careful navigation, as excessive reliance on extrinsic rewards in digital environments may inadvertently undermine intrinsic motivation (Deci and Ryan, 2013). Furthermore, the rapid evolution of digital technologies frequently outpaces pedagogical research, creating a persistent gap between technological possibilities and evidence-based implementation strategies.

Video-based learning has emerged as a particularly promising component of effective BL in PE, with a growing body of research identifying optimal design principles. Duration represents a critical factor, with empirical studies demonstrating superior engagement and learning outcomes for videos under 6 min, and particularly those less than 3 min for skill-based tutorials (Guo et al., 2014; Brame, 2017). Production style significantly impacts effectiveness, with “Khan-style” tablet drawings and instructor-present formats proving most beneficial for retention (Guo et al., 2014). Neurocognitive evidence confirms that well-designed video instruction enhances PE outcomes through three synergistic mechanisms: temporal segmentation (Mayer and Chandler, 2001) manages working memory constraints while modality-specific presentation (Mayer, 2014) optimally pairs visual demonstrations with auditory explanations. This dual coding approach (Paivio, 2013; Mayer, 2024) creates complementary verbal and visual memory traces that simultaneously improve biomechanical understanding and skill retention. The inherent self-paced review capability (Ghorbel et al., 2024) further strengthens this process by allowing repeated access to content, enabling deeper cognitive processing and more robust memory consolidation. Important limitations must be considered, including the potential for over-reliance on extrinsic feedback to impede intrinsic skill correction mechanisms (Ghorbani et al., 2020; Maslovat et al., 2010) and the disengaging effects of poorly paced or excessively lengthy content (Lee et al., 2021). While evidence-based design principles enhance the efficacy of video-based learning in PE, the integration of social media platforms, leveraging ultra-short formats and interactive features, presents an untapped opportunity to further optimize engagement, community building, and skill acquisition.

The emergence of social media platforms has introduced transformative possibilities for PE instruction, though their formal educational potential remains largely untapped. TikTok’s remarkable success in informal learning contexts demonstrates the power of ultra-short video formats (<1 min) combined with interactive features like hashtags and duets for knowledge sharing and community building (Liu, 2022). Similarly, Facebook’s collaborative tools–including groups, live streaming, and feedback mechanisms–align with Social Presence Theory (Short et al., 1976) to extend learning communities beyond physical classroom boundaries (Lacka et al., 2021). These platforms capitalize on fundamental neurocognitive processes; for instance, the Mirror Neuron System (MNS) activates most effectively when learners observe movements within their achievable skill range, facilitating embodied learning and skill acquisition (Calvo-Merino et al., 2006). Moreover, research on attentional focus demonstrates that external cues (e.g., “In a handstand, lock your shoulders, engage your core, and push upward while keeping your body perfectly aligned”) consistently produce superior performance outcomes compared to internal cues (Wulf, 2013), a principle that can be effectively incorporated into digital instruction. Recent research has explored the “Add-on” model, an instructional strategy that enriches core educational content through supplementary, non-disruptive enhancements. Trabelsi, Ghorbel (Trabelsi et al., 2025) demonstrated its effectiveness in higher education by integrating *Did You Know* (DYK) segments with static images, showing improved knowledge assimilation. Furthermore, a significant research gap persists regarding the implementation of short-form video adaptations in secondary PE contexts. This study introduces and systematically evaluates the video-based “Did You Know?” (DYKV) model, an innovative BL framework that synthesizes these evidence-based elements into a cohesive approach for gymnastics instruction. In Tunisia, gymnastics holds significant importance within the national PE curriculum (Amade-Escot et al., 2015). However, the discipline faces substantial implementation challenges, including inadequate infrastructure, insufficient allocated instructional time, and technical complexity requiring extended learning periods. The DYKV model represents three significant advances in PE pedagogy: (1) it provides the first structured framework for integrating ≤30-s instructional videos into secondary PE curricula. These short-form videos are inherently engaging due to their brevity and visual appeal, which can more effectively capture student attention (Yan and Yan, 2024; Žufić and Bodiš, 2023), (2) it leverages the untapped educational potential of mainstream social media platforms as formal learning tools, and (3) it applies neuroscientific principles of MNS activation through video information to enhance skill acquisition. Grounded in Dual Coding Theory (Paivio, 2013) and Social Presence Theory (Short et al., 1976), the model combines visual demonstrations, auditory explanations, and textual information through Facebook’s interactive platform, creating a multimedia learning environment that supports diverse learning preferences.

This study examines three primary hypotheses derived from this theoretical and empirical foundation. First, we hypothesize that students in the DYKV experimental group will demonstrate significantly greater improvements in gymnastics skill performance compared to those receiving traditional instruction (i). Second, we predict that exposure to the DYKV model will increase participants’ autonomous motivation (ii). Third, we anticipate that controlled motivation levels will remain stable across the intervention period (iii). These hypotheses will be tested through a rigorous experimental design employing standardized skill assessments and validated motivation measures.

## Methods

### Participants

We calculated the required sample size using G*Power V.3.1.9.6 (Faul et al., 2009) to ensure adequate statistical power for our two-way mixed ANOVA design. A sensitivity analysis was conducted to determine the minimum effect size that could be reliably detected given our planned parameters: an alpha level of 0.05, a desired power of 0.80, and the structure of the mixed design. Based on this analysis, the effect size f was determined to be 0.20. Accordingly, the required total sample size was calculated to be 52 participants. To ensure robustness, initially, 65 students were assessed for eligibility, of whom 60 met the inclusion criteria. After a baseline assessment evaluating motor performance, knowledge, and motivation, exclusion criteria were applied, resulting in a final sample of 54 participants (*n* = 54; boys = 24; girls = 30) whose data were analyzed. The participants, with a mean age of 17 years (SD = 0.4), were second-year secondary education students from an educational institution in Sfax, Tunisia. The sample was divided into a control group (TA; *n* = 27; 13 boys, 14 girls) and an experimental group (DYKV; *n* = 27; 11 boys, 16 girls).

### Legal and ethical considerations

Prior to the commencement of the study, all necessary legal authorizations were obtained from relevant stakeholders, including the head of the Regional Commission for Education (Sfax II), the principal of the “Monji Slim” secondary school (the study site), and the participating teacher. Parents of underaged students were provided with an informed consent letter outlining the study’s procedures in detail. By signing the letter, they granted permission for their children to participate in the research. Students of legal age independently signed their own consent forms. The research protocol was reviewed and approved by the local Personal Protection Committee (CPP SUD N^o^ 0551/2023), ensuring adherence to ethical standards for educational research as outlined by the British Educational Research Association (BERA, 2018).

### Experimental setup

Due to the school administration’s restrictions on altering the composition of the two available classes, conducting randomized controlled trials was not feasible. Consequently, a quasi-experimental design was employed. The two classes were randomly assigned to either a control group or an experimental group, with each class representing one group. The first class, designated as the experimental group, was labeled the “video-based “Did You Know?” (DYKV) group,” while the second class, serving as the control group, was labeled the “Traditional Approach (TA) group.”

**Figure 1:**
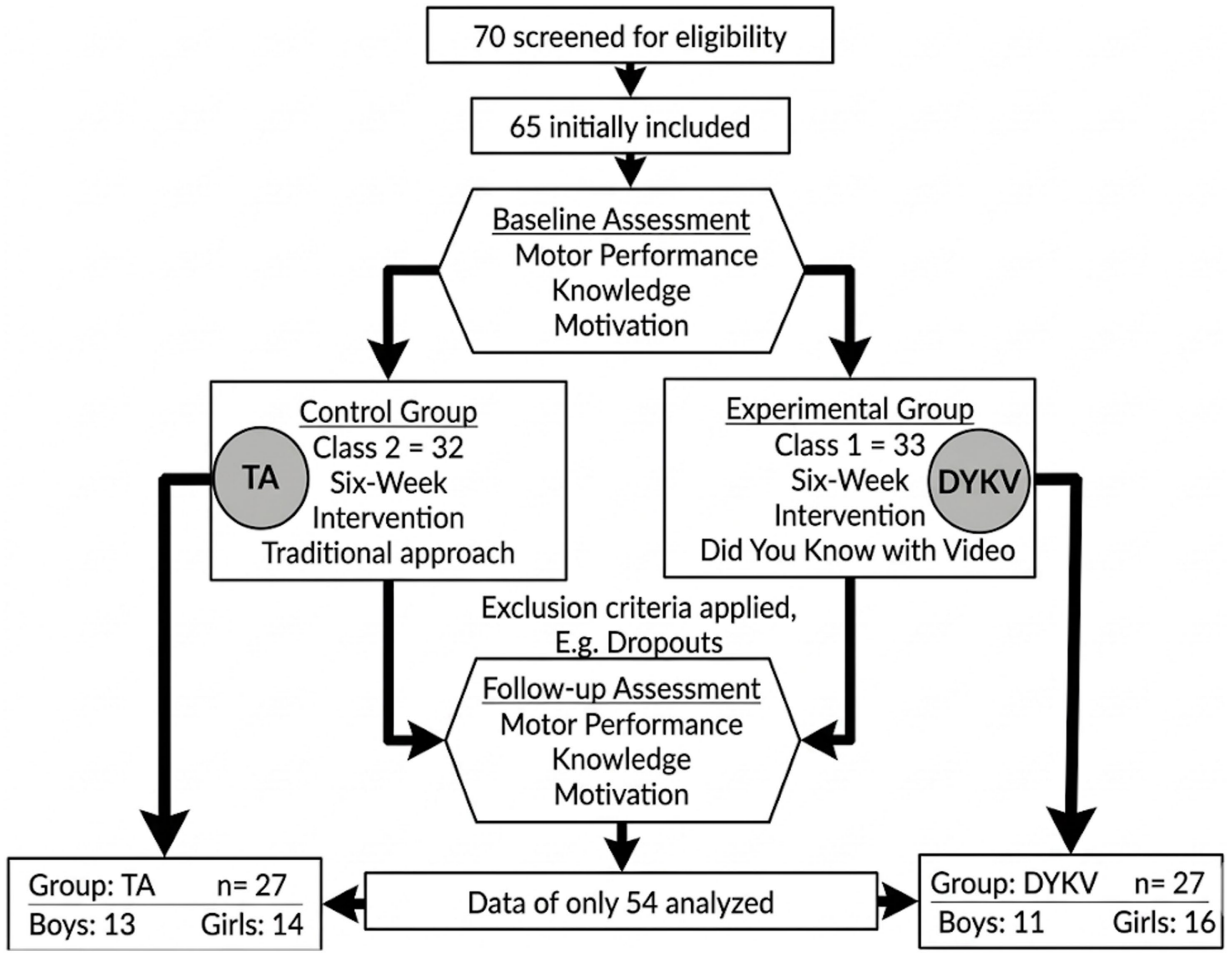
Study flowchart: experimental design and participant progression.

As previously noted, following the exclusion of certain participants, data from 14 girls and 13 boys (*N* = 27) in the TA group and 11 girls and 16 boys (*N* = 27) in the DYKV group were included in the final analysis, resulting in a total sample size of 56. The experimental design is depicted in “Figure 1.”

### Study sessions

The TA group participated in six PE sessions scheduled on Tuesday from 10:00 a.m. to 12:00 p.m., while the DYKV group attended their six sessions on Friday during the same time slot. Each two-hour session followed a structured timeline: the first 30 min were allocated for gymnasium setup by the research team and teacher orientation. This was followed by a 10-min opening that included safety checks and initial briefings, a 15-min warm-up, a 60-min main instructional segment, and a 5-min cool-down period.

The main instructional segment of each session was organized around four learning tasks, each lasting 10 min, with a 5-min recovery break between the second and third tasks. This was followed by a 12-min final assessment task, preceded by a 3-min recovery break. These tasks were dedicated to specific gymnastics skills, as detailed in Table 1. Each 10-min task commenced with the teacher providing instructions, demonstrating the skill, and explaining execution methods and success criteria. Ample time was then devoted to practicing the skill (repetitions), during which teachers offered constructive feedback. Time allocation during learning tasks may have varied slightly between groups due to differences inherent to the experimental conditions.

**Table 1:** Gymnastics skills that were taught during each session of the study.

Learning session	Gymnastics skills		Skill complexity
	For males	For females	
Session 1	Salute (masculine) Front scale ½ jump turn Full jump turn	Salute (feminine) Arabesque ½ jump turn Full jump turn	Low
Session 2	Front. side and back support Forward roll Backward roll		Low to Medium
Session 3	Straddle forward roll Straddle backward roll Dive roll		Medium to High
Session 4	Handstand		High
Session 5	Cartwheel		High
Session 6	Roundoff		High

The PE sessions were conducted in the gymnasiums of the secondary school hosting the study. The sessions took place under North African climatic conditions, with indoor temperatures ranging between 16 °C and 19 °C during the study period.

To ensure safety, shock-absorbent mats (152 × 304 × 25 cm) were used to protect students from falls and slipping, fostering confidence in performing complex skills such as handstands and cartwheels. For beginners practicing forward and backward rolls, semi-flexible incline mats (81 × 142 × 55 cm) provided additional protection for the head, neck, and spine, emphasizing safety during initial learning stages.

### Intervention procedures

#### The traditional approach group

The control group participated in a six-week intervention during which they received instruction following the teacher’s traditional teaching method in their PE classes. This method predominantly relied on verbal explanations and instructions for the learning tasks, with peers demonstrating gymnastic movements to facilitate skill acquisition. Feedback was provided directly by the teacher, but no technology-enhanced interventions were integrated into the instructional process.

#### The video-based “Did You Know?” model group

**Figure 2:**
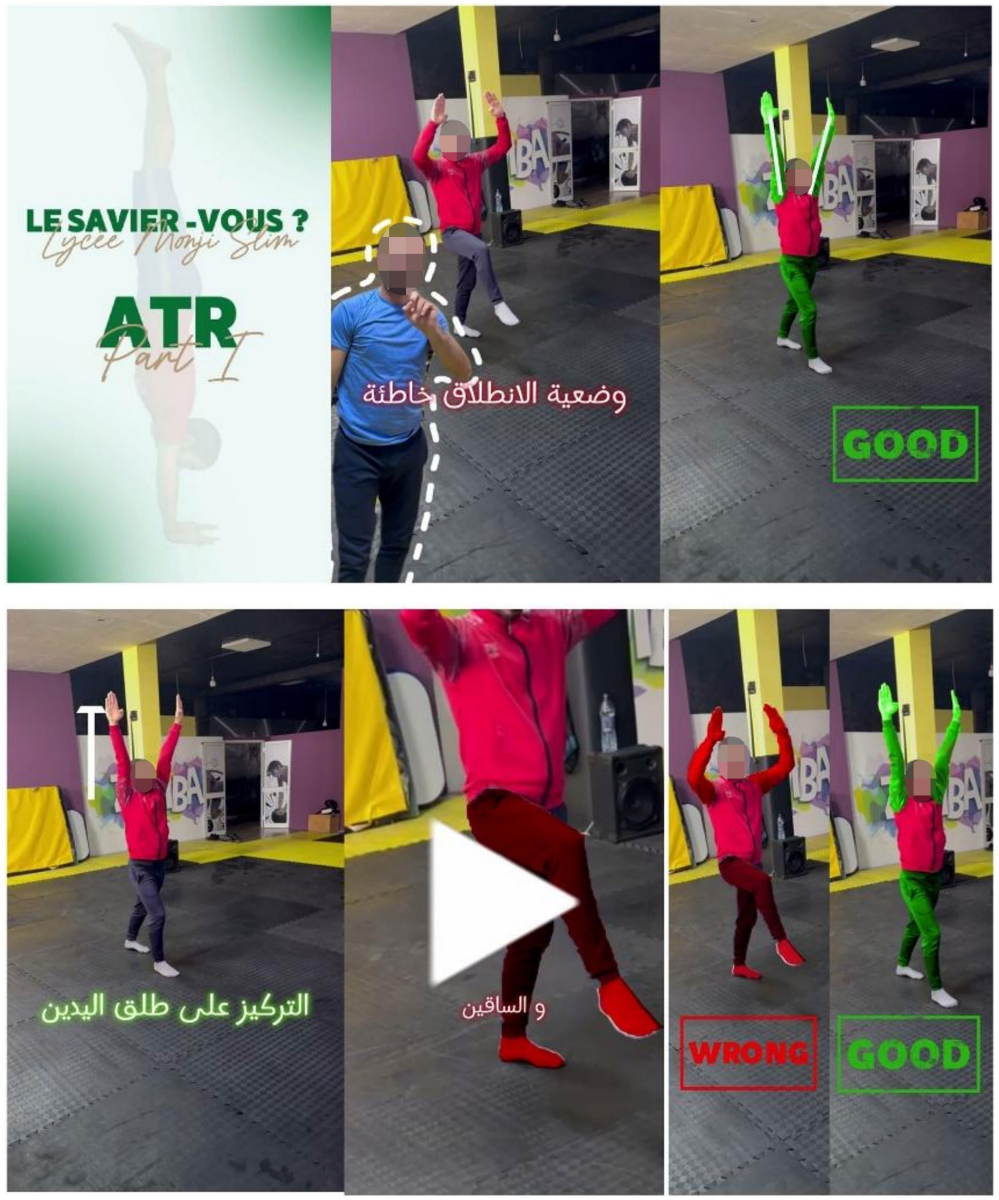
Screenshots from the “Did You Know?” videos used in the study. Example videos used in the study are available at: https://drive.google.com/drive/folders/1o4_1GJdaRaP3ifaUSoZzWaHdUazB7ZiY?usp=sharing.

Participants in the experimental group engaged in a learning unit designed around the DYKV model. This approach provided them with unrestricted access to a dedicated Facebook group, available both before and after the scheduled classes. The group served as a platform where participants could view and interact with posts containing short, instructional videos related to specific gymnastics elements, accompanied by detailed success criteria for each skill (Figure 2). These resources were carefully curated to enhance participants’ understanding and support their mastery of gymnastic techniques.

The DYKV intervention was delivered via a private Facebook group in the form of dynamic reels (approx. 30 s each). Depending on the technical requirements of the gymnastics skills taught in the PE class for that specific week, 2–3 reels were published. This ensured that the digital content was directly synchronized with the practical curriculum. To monitor adherence, the researchers used the “Seen by” feature on Facebook, confirming that all participants had viewed the instructional material prior to the face-to-face sessions. This multimedia resource provided a technical demonstration of the movement, supplemented with critical performance indicators: (1) optimal body positioning, (2) sequential execution phases, and (3) corrective feedback for common errors. The video-based material served a dual pedagogical purpose: reinforcing theoretical knowledge while facilitating skill transfer to practical execution. Posts adhered to a standardized design protocol, combining concise textual explanations with dynamic visual aids (e.g., motion diagrams, annotated still frames) to enhance cognitive engagement.

#### Teacher orientation

The study was conducted by an experienced female PE teacher (age 39) with specialized training in gymnastics and 13 years of secondary school teaching experience. Assigned to both classes by the school administration prior to the study, she was already familiar to all participants, ensuring students were comfortable with her established teaching style. To maintain consistency between groups, the teacher followed standardized protocols. Before each session, she received 30 min of training emphasizing equitable instruction - providing equal demonstration quality, feedback quantity, and attention distribution between the experimental (DYKV) and control groups. These measures effectively controlled for potential instructor bias, ensuring any observed differences could be attributed to the instructional methods rather than teacher-related variables. The approach strengthened the study’s internal validity while maintaining authentic classroom conditions.

### Data collection

As depicted in Figure 1, in addition to six main experimental sessions, two 2-h sessions were conducted for pre- and post-testing, aimed at assessing motor learning, knowledge retention, and motivation.

#### Motor learning in gymnastics

The effectiveness of the teaching approach on students’ motor learning was evaluated by comparing their performance in gymnastics floor routines conducted before and after the intervention for each group. The floor routine, designed by three PE teachers with expertise in gymnastics, included all the skills to be taught (as outlined in Table 1). Prior to both the pretest and posttest, students in both the TA and DYKV groups viewed a video of the routine three times. This video provided a visual demonstration of the required skills to assist students, particularly those unsure of how to proceed during the assessment. Collective video viewing took place in the gymnasium using an Epson EB-972 projector connected to a Lenovo ThinkPad T470s laptop.

For both assessments, four gymnastics experts served as judges. They evaluated students’ performances based on routine execution, neatness, and technical accuracy, following criteria established in an assessment grid that they had collaboratively developed and agreed upon beforehand. Starting from a base score of 20 points, deductions were made for errors based on their severity: minor errors (0.5 points), serious errors (1–2 points), and eliminatory errors (2–4 points), with the complexity of the skill influencing the deductions (refer to Table 2). Each judge recorded observed errors live using the assessment grid and a scoring sheet, subsequently determining the corresponding deductions after the routine’s completion. Final scores for each student were calculated as the average of the judges’ scores.

**Table 2:** Comprehensive scoring scheme for the gymnastics floor routine based on skill complexity and error severity.

Skill	Skill complexity	Deducted Points		
		Minor error	Serious error	Eliminatory error
Salute	Low	0.5	1	2
Scale/Arabesque	Low	0.5	1	2
Turning jumps	Low	0.5	1	2
Support skills	Low	0.5	1	2
All forward rolls	Medium	0.5	1.5	3
All backward rolls	Medium	0.5	1.5	3
Dive Roll	Medium	0.5	1.5	3
Handstand	High	0.5	2	4
Cartwheel	High	0.5	2	4
Roundoff	High	0.5	2	4

To ensure the reliability of the assessment, inter-rater agreement among the judges was measured using Fleiss’ Kappa. The calculated Kappa values were 0.84 for the pretest and 0.91 for the posttest, indicating a high level of agreement among the judges at both evaluation stages. These results demonstrate the consistency and reliability of the assessment process.

#### Gymnastics-specific knowledge retention

Students’ gymnastics-specific knowledge was assessed before and after the practical intervention using a custom-designed written test. This test was developed based on content from French gymnastics manuals (Kacou, 2015; Ritorto, 2007) and refined through consultations with experts, including gymnastics coaches and PE teachers. The test was aligned with the Tunisian Official Directives for PE (MJES, 1990) to ensure its relevance and adherence to curricular standards.

The final test, presented in French (the language of instruction) with Arabic subtitles (students’ mother tongue), consisted of 10 multiple-choice questions covering topics such as skill names, technical execution, and sequence structuring. Each correct response contributed to a total score of 20 points (2 points per question). To discourage random guessing, incorrect selections resulted in the deduction of potential points for the corresponding question. Some questions included multiple correct answers, requiring students to identify all correct options to receive full credit.

#### Motivation for gymnastics

The Situational Motivation Scale (SIMS), developed by Guay et al. (2000), is a widely recognized instrument for evaluating educational motivation, particularly for measuring controlled and autonomous motivation. In our study, the SIMS was utilized to assess these two types of motivation within the context of gymnastics before and after the experimental phase.

The SIMS prompts participants with the question, “Why are you currently engaged in this activity?” and provides 16 potential response options (items). Students were asked to rate each item on a scale from 1 (not at all) to 7 (exactly). The scale categorizes motivation into four types: intrinsic motivation (items 1, 5, 9, and 13), identified regulation (items 2, 6, 10, and 14), external regulation (items 3, 7, 11, and 15), and amotivation (items 4, 8, 12, and 16). An autonomous motivation index was derived by averaging the responses for intrinsic motivation and identified regulation (Schulte-Uentrop et al., 2020). Similarly, a controlled motivation index was calculated by averaging the responses for external regulation and amotivation (Schulte-Uentrop et al., 2020).

Given that the original version of the SIMS was in English (Guay et al., 2000), we undertook a cross-cultural adaptation process 5 months prior to the study’s commencement. This process involved multiple rounds of forward and backward translation to French and Arabic by a panel of five trilingual experts until consensus was achieved. The Content Validity Index (CVI) for individual items averaged 0.89, and the scale-level CVI was 0.92, confirming the content validity of the translated instrument.

The adapted version of the SIMS, presented in French with Arabic subtitles, was piloted with 27 secondary school students. The internal consistency of the scale was found to be robust, with Cronbach’s alpha coefficients ranging from 0.81 to 0.86 for the various factors (intrinsic motivation: 0.84; identified regulation: 0.81; external regulation: 0.86; amotivation: 0.84). Furthermore, test–retest reliability was established with an intraclass correlation coefficient of 0.79, measured 28 days after the initial test.

### Data analyses

Due to the non-normal distribution of many score datasets, as confirmed by the Shapiro–Wilk test (*p* < 0.05), and the violation of the homogeneity of variance assumption, as indicated by Levene’s test (*p* < 0.05), non-parametric statistical methods were utilized for data analysis. To compare the two groups with repeated measures (pre-test and post-test) based on their pedagogical approaches, the Aligned Rank Transform (ART) test was applied. This method is particularly effective for analyzing repeated measures data when normality and homogeneity assumptions are not met.

The effect size for the ART test was computed using a formula analogous to partial eta-squared (η_p_^2^), where η_p_^2^ = $\frac{\mathrm{SS}\text{total}}{\mathrm{SS}\text{effect}\;}$ . Here, SSeffect represents the sum of squares for the effect, and SStotal denotes the total sum of squares. Effect sizes were categorized as small (η_p_^2^ < 0.06), moderate (0.06 ≤ η_p_^2^ < 0.14), or large (η_p_^2^ ≥ 0.14). For post-hoc Wilcoxon signed-rank tests, the effect size *r* was calculated using $r=\frac{z}{\surd n}$ , where Z is the standardized test statistic and N is the total number of observations. Effect sizes for r were interpreted as small (*r* < 0.3), moderate (0.3 ≤ *r* < 0.5), or large (*r* ≥ 0.5).

Motivation scores, including autonomous and controlled motivation, were analyzed using the Wilcoxon test to compare pre- and post-test results for each group, with statistical significance set at *p* < 0.05. Effect sizes were calculated using $r=\frac{z}{\surd n}$ , with thresholds for small, medium, and large effects defined as 0.1, 0.3, and 0.5, respectively (Cohen, 1988).

Data are presented as mean ± standard deviation (SD) in the text, while figures display mean ± standard error (SE). All statistical analyses were performed using R software (Version 4.3.1, R Foundation for Statistical Computing, Vienna, Austria).

## Results

### Motor learning in gymnastics

The results of the Aligned Rank Transform (ART) analysis revealed a significant main effect of “test time” [*F*_(1,52)_ = 123.38; *p* < 0.001], with a very strong effect size (η_p_^2^ = 0.70) and full statistical power. Furthermore, the interaction effect between “group” and “test time” was also significant [*F*_(1,52)_ = 5.53; *p* < 0.05], demonstrating a medium effect size (η_p_^2^ = 0.124).

As shown in Figure 2, both the TA group (pretest: 9.12 ± 4.13 points; posttest: 12.81 ± 2.81 points; *p* < 0.001) and the DYKV group (pretest: 7.38 ± 4.04 points; posttest: 13.05 ± 4.30 points; *p* < 0.001) exhibited significant improvements in floor routine scores. The significant “group × test time” interaction indicates that the DYKV group achieved greater improvement compared to the TA group from pretest to posttest (*p* < 0.05). Specifically, the mean change for the DYKV group was ΔDYKV = 5.66 ± 2.72 points (76.69%), while the TA group showed a mean change of ΔTA = 3.43 ± 3.42 points (37.58%), corresponding to a medium effect size (η_p_^2^ = 0.096).

### Gymnastic-specific knowledge retention

The Aligned Rank Transform (ART) analysis revealed a significant main effect of “test time” [*F*_(1,52)_ = 59.24; *p* < 0.001], with a substantial effect size (η_p_^2^ = 0.51) and complete statistical power. Furthermore, the interaction between “group” and “test time” was also significant [*F*_(1,52)_ = 5.58; *p* < 0.05], indicating a medium effect size (η_p_^2^ = 0.096) and a statistical power of 64%.

**Figure 3:**
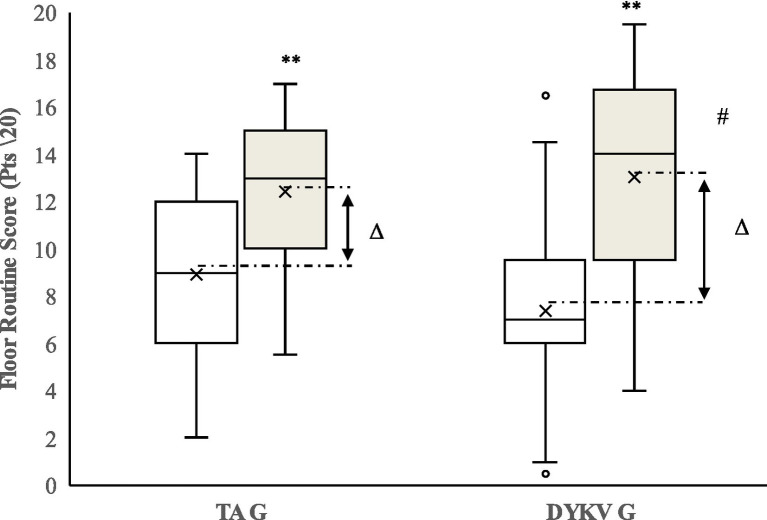
Comparison of mean floor routine scores (±SE) between the traditional approach (TA) and the video-based “Did You Know?” (DYKV) groups across test times (pre to posttest). ^**^p < 0.001: significantly different from pre-test. #*p* < 0.05: significant difference compared to TA group.

**Figure 4:**
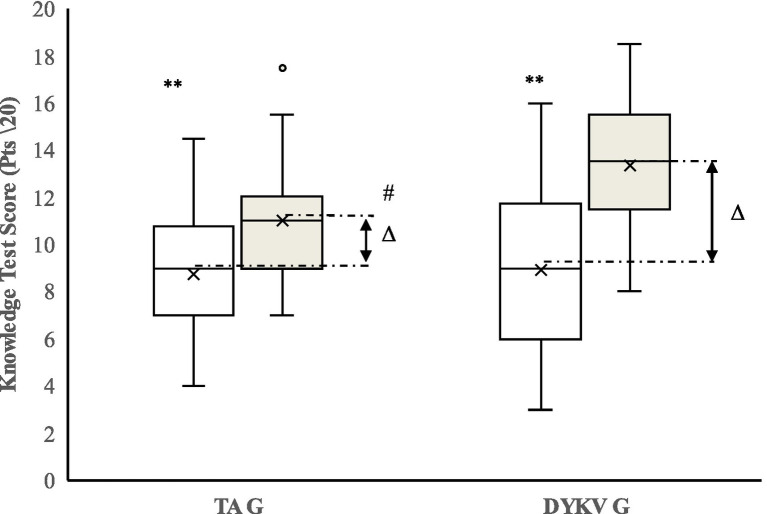
Comparison of knowledge test score (±SE) between the traditional approach (TA) and the video-based “Did You Know?” (DYKV) groups across test times (pre to posttest). * *p* < 0.001: significantly different from post-test. # *p* < 0.05: significant difference compared to DYKV group.

As shown in Figure 3, both the TA group (pretest: 8.72 ± 3.67 points; posttest: 11.03 ± 2.54 points; *p* < 0.01) and the DYKV group (pretest: 8.90 ± 3.29 points; posttest: 13.35 ± 2.85 points; *p* < 0.001) demonstrated significant improvements in their gymnastic-specific knowledge test scores. The significant “group × test time” interaction suggests that the DYKV group experienced a more pronounced improvement compared to the TA group from pretest to posttest (*p* < 0.05). Specifically, the DYKV group showed a mean increase of ΔDYKV = 4.44 ± 3.26 points (49.89%), which was significantly greater than the TA group’s increase of ΔTA = 2.31 ± 3.35 points (26.53%) (*p* < 0.05). This difference corresponds to a medium effect size (ηp^2^ = 0.096) (Figure 4).

### Motivation for gymnastics

#### Autonomous motivation index (AMI)

In the DYKV group, a statistically significant increase in the AMI (*z* = 3.84; *p* < 0.001) was observed, reflecting a large effect size (*r* = 0.73). Specifically, the mean AMI score in the DYKV group increased from 3.96 ± 1.27 points prior to the intervention to 5.14 ± 0.94 points post-intervention, measured on a 7-point Likert scale. Conversely, in the TA group, a significant decrease in the mean AMI (*z* = 3.33; *p* < 0.001) was noted, with scores declining from 4.34 ± 1.15 points to 3.03 ± 1.31 points on the same scale. This reduction was associated with a large effect size (*r* = 0.64). These findings are visually represented in “Table 3.”

**Table 3:** Comparison of mean autonomous motivation indexes before and after the intervention.

Group	Pretest (Mean ± SD)	Posttest (Mean ± SD)	*p*
DYKV Group	3.96 ± 1.27	↑ 5.14 ± 094	<0.001
Control Group	4.34 ± 1.15	↓ 3.03 ± 1.31	<0.001

↑: increased after the intervention. ↓: decreased after the intervention.

#### Controlled motivation index (CMI)

In the DYKV group, a statistically significant decrease in the CMI (*z* = 4.36; *p* < 0.001) was observed, indicating a large effect size (*r* = 0.87). Specifically, the mean CMI score in the DYKV group declined from 4.51 ± 0.88 points before the intervention to 2.98 ± 1.23 points post-intervention, measured on a 7-point Likert scale. In contrast, the TA group exhibited a significant increase in the mean CMI (*z* = 3.34; *p* < 0.001), with scores rising from 3.89 ± 1.28 points to 4.88 ± 1.05 points on the same scale. This increase was associated with a large effect size (*r* = 0.64). These outcomes are visually depicted in “Table 4.”

**Table 4:** Comparison of mean controlled motivation indexes (±SD) after the traditional approach (TA) intervention and the DYKV intervention.

Group	Pretest (Mean ± SD)	Posttest (Mean ± SD)	*p*
DYKV Group	4.51 ± 0.88	↓ 2.98 ± 1.23	<0.001
Control Group	3.89 ± 1.28	↑ 4.88 ± 1.05	<0.001

↑: increased after the intervention. ↓: decreased after the intervention.

## Discussion

This study investigated the impact of the DYKV model, a BL approach integrating educational videos and a collaborative platform (Facebook). The DYKV model aimed to enhance motor performance, knowledge retention and motivation compared to traditional teaching methods in gymnastics.

A key finding is that students exposed to the DYKV model demonstrated significantly greater improvement in gymnastics routine performance compared to those receiving traditional instruction. While this study is among the first to examine the impact of the DYKV model in secondary school settings, it integrates video-based learning and social media within a BL approach. Similar Research (Garcia, 2024) suggests that TikTok effectively enhances engagement with fitness content and facilitating practical application of exercise. However, our results differ from earlier studies, such as those by Goodyear et al. (2019), which indicate that young individuals often skip or ignore content related to physical activity, nutrition, and body image, limiting their opportunities to improve their understanding of movement acquisition. The discrepancy may stem from differences between content presentation (Trabelsi et al., 2025).

The improved gymnastics performance in the DYKV group is supported by better retention of gymnastics-specific knowledge. Research on motor learning and neuroplasticity, such as Dayan and Cohen (2011), posit that motor skill development entails neural activity modifications across various cerebral regions, including but not limited to the dorsolateral prefrontal cortex, primary motor cortex, and supplementary motor area. These changes suggest the recruitment of additional cortical substrates with practice, optimizing neural efficiency as motor skill acquisition advances (Dayan and Cohen, 2011). The MNS, which is essential for observational learning, enables the internal simulation of observed actions, facilitating their understanding and reproduction (Van Gog et al., 2009; Ray, 2009). Although still maturing in adolescents, the MNS is sufficiently developed to support the acquisition of motor skills through video observation, as demonstrated by recent research on action observation networks in this population (Lesourd et al., 2023). In this study, the use of short (30-s), targeted videos (Liu, 2022), combined with visual and auditory explanations, likely enabled students to better understand and memorize gymnastic techniques. This approach is supported by Mayer (2002), who demonstrated that integrating visual and verbal elements in educational videos enhances comprehension and information retention. Infographics and visual demonstrations likely helped students internalize technical concepts more effectively. Ibrahim et al. (2012) found that segmented and well-structured videos enhance knowledge retention by reducing cognitive load. These posts typically present concise pieces of information accompanied by engaging visuals or graphics, designed to capture attention and stimulate curiosity among users. For example, consider a video that depicts an illustrated diagram showcasing diverse gymnastic movements. Alongside this visual representation is the statement: “Did You Know? Handstand requires the body to be aligned and well-engaged. This example of pairing visual and factual elements is designed to captivate viewers, deepening understanding of gymnastic concepts. By providing access to video sharing in the Facebook group, students are afforded the opportunity to engage with the content at their own pace and preferred time. This flexibility in not only primes students with foundational knowledge but also allows them to revisit and reinforce technical knowledge content as needed, thereby fostering deeper understanding and retention (Topping et al., 2022). Social Presence Theory (Short et al., 1976) to extend learning communities beyond physical classroom boundaries (Lacka et al., 2021). These platforms capitalize on fundamental neurocognitive processes; for instance, the MNS activates most effectively when learners observe movements within their achievable skill range, facilitating embodied learning and skill acquisition (Calvo-Merino et al., 2006).

This study also revealed intriguing findings concerning enhanced motivation levels towards PE classes, particularly in gymnastics, when the DYKV model is implemented. The results demonstrated that autonomous motivation increased among students exposed to the DYKV model but decreased under traditional teaching methods. Conversely, controlled motivation decreased with the DYKV model but increased with traditional teaching methods. The divergent trends observed in autonomous and controlled motivation lend credibility to the self-reported data, as their opposition aligns logically; as autonomous motivation increases, controlled motivation must decrease (Sandrin et al., 2022). These findings further consolidate previous research proposed by Standage and Gillison (2007) demonstrated that autonomy-supportive learning environments increase intrinsic motivation and student engagement in PE. The Facebook platform, with its interactive and collaborative features, likely created a sense of community and engagement, encouraging students to take an active role in their learning. This observation is supported by Akcaoglu and Lee (2018), who found that Facebook groups promote social presence and interaction among students, thereby enhancing motivation and engagement. Furthermore, Vanek et al. (2018) highlighted that social media platforms like Facebook enable students to share resources, receive feedback, and collaborate, reinforcing their sense of competence and belonging. The synergy between improved knowledge acquisition and heightened motivation appears to be responsible for the enhanced gymnastics performance demonstrated by students following the DYKV model. This study’s mixed-methods approach provided valuable data on student acceptance of technology-enhanced learning, offering further evidence of the model’s effectiveness.

The decision to utilize Facebook, currently the world’s most popular social media platform (Kepios, 2024), proved particularly strategic. Unlike conventional learning management systems (LMS) that students often find challenging to navigate (Butt et al., 2021), Facebook’s familiar interface and group functionality likely played a key role in the intervention’s positive outcomes (Xu et al., 2024). Research by Manca and Ranieri (2016) supports this approach, characterizing Facebook as a more accessible and flexible platform compared to traditional LMS. While this study does not provide definitive data on Facebook’s specific impact, numerous studies confirm its effectiveness in supporting asynchronous learning environments (Todorovic et al., 2021), reinforcing its value in educational contexts. While the improvements in motor performance and knowledge are consistent with the Mirror Neuron System and Cognitive Load Theory, it is important to note that these mechanisms remain theoretical inferences in the current study. We did not empirically track the specific frequency of video views or the nature of digital interactions on the Facebook platform.

While the DYKV model demonstrates clear pedagogical benefits, its implementation via social media necessitates a careful analysis of potential risks. Using platforms like Facebook introduces the possibility of information interference from advertisements or non-educational notifications, which may distract students. To mitigate this, the intervention utilized a “Closed Private Group” to filter external interactions. Furthermore, to address concerns regarding screen dependence, the DYKV videos were intentionally designed to be short (under 60 s), focusing on “micro-learning” rather than prolonged screen time. Regarding privacy and risk control, the study leveraged the robust security infrastructure of Facebook, a globally recognized platform that adheres to stringent international data protection standards (Xu et al., 2024). To ensure a safe learning environment, the DYKV model was implemented through ‘Private Groups,’ where the teacher acted as the sole administrator. This setting ensures that all content and interactions remain invisible to the public and that only verified students are granted access. By utilizing these established privacy frameworks, the DYKV model provides a secure digital space that protects student data while remaining highly accessible.

### Practical recommendations

The findings of this study underscore important pedagogical implications for gymnastics instruction in PE. First, the integration of digital resources, such as instructional videos and collaborative platforms, provides an effective strategy to address time constraints and facilitate individualized learning. By extending the learning process beyond the physical classroom, these tools offer students greater flexibility to review content, reinforce understanding, and engage in self-regulated learning.

Second, the observed positive impact on motivation suggests that interactive and student-centered learning environments may contribute to more engaging and sustainable learning experiences. Given the increasing emphasis on technology-enhanced and BL approaches in education, these findings advocate for the adoption of hybrid models that integrate face-to-face instruction with digital resources to maximize learning outcomes.

While this study demonstrated the effectiveness of the DYKV in gymnastics, its applicability could extend to other areas of PE, such as athletics, team sports, or dance. Future research should explore these possibilities, as well as examine the long-term effects of BL on motivation, skill retention, and continued engagement in physical activities. Moreover, further investigations could assess the impact of different digital platforms and video formats (e.g., interactive videos, augmented reality simulations) on learning effectiveness.

### Research limitations

Despite the valuable insights provided by this study, few limitations should be acknowledged. First, the sample consisted of 54 students aged 17 from a single secondary school in Sfax, Tunisia. Consequently, the results may not be fully representative of different age groups, such as primary school pupils or university students, nor can they be automatically generalized to different cultural or educational systems. Future research should employ larger, multi-centric samples across diverse geographical regions and age groups to test the robustness of the DYKV model in varying pedagogical contexts. Second, Furthermore, certain baseline characteristics that could act as confounding variables (such as students’ prior gymnastics training experience, the quality of their home internet access, and their daily habits regarding social media use) were not formally measured or included as covariates in the statistical analysis. While the study controlled for teaching hours and pedagogical leadership (same teacher for both groups), the absence of these individual background variables limits our ability to fully isolate the causal impact of the DYKV model. Future studies should employ more comprehensive baseline screening to include these factors as covariates (e.g., using ANCOVA) to provide a more nuanced understanding of the intervention’s efficacy. Third, the study was conducted over a limited time period, and the long-term effects of the BL model on motor learning, knowledge retention, and motivation remain unclear. Future research could explore the sustainability of these effects over extended durations. Fourth, the study focused solely on gymnastics, and while the results may be applicable to similar PE contexts, further studies are needed to determine whether the findings can be replicated across different sports or educational settings. Lastly, the intervention sessions were conducted on different weekdays due to the existing school schedule. This may have introduced contextual variables, such as varying levels of student fatigue or academic stress prior to the PE sessions. However, both groups attended their sessions during the morning blocks to control for significant circadian fluctuations. Future research should aim to balance session timings across weekdays to further isolate the impact of the blended learning model from these environmental factors.

## Conclusion

Overall, this study provides strong evidence that integrating BL models in PE can significantly enhance motor skill acquisition, knowledge retention, and autonomous motivation. By leveraging instructional videos and collaborative online platforms, educators can create more inclusive, flexible, and engaging learning environments. While these findings are promising, further research is needed to evaluate their long-term implications and adaptability to diverse PE settings. Given the increasing integration of technology in education, these results highlight the potential of BL to transform traditional PE practices and promote lifelong engagement in physical activity.

## Data Availability

The raw data supporting the conclusions of this article will be made available by the authors, without undue reservation.
